# Novel Bioimaging Techniques of Metals by Laser Ablation Inductively Coupled Plasma Mass Spectrometry for Diagnosis Of Fibrotic and Cirrhotic Liver Disorders

**DOI:** 10.1371/journal.pone.0058702

**Published:** 2013-03-07

**Authors:** Pornwilard M-M, Ralf Weiskirchen, Nikolaus Gassler, Anja K. Bosserhoff, J. Sabine Becker

**Affiliations:** 1 Institute of Clinical Chemistry and Pathobiochemistry, RWTH University Hospital Aachen, Aachen, Germany; 2 Central Division of Analytical Chemistry, Forschungszentrum Jülich, Jülich, Germany; 3 Department of Chemistry and Center for Innovation in Chemistry, Mahidol University, Bangkok, Thailand; 4 Institute of Pathology, RWTH University Hospital Aachen, Aachen, Germany; 5 Institute of Pathology, University Hospital of Regensburg, Regensburg, Germany; University of Navarra School of Medicine and Center for Applied Medical Research (CIMA), Spain

## Abstract

**Background and Aims:**

Hereditary disorders associated with metal overload or unwanted toxic accumulation of heavy metals can lead to morbidity and mortality. Patients with hereditary hemochromatosis or Wilson disease for example may develop severe hepatic pathology including fibrosis, cirrhosis or hepatocellular carcinoma. While relevant disease genes are identified and genetic testing is applicable, liver biopsy in combination with metal detecting techniques such as energy-dispersive X-ray spectroscopy (EDX) is still applied for accurate diagnosis of metals. *Vice versa*, several metals are needed in trace amounts for carrying out vital functions and their deficiency due to rapid growth, pregnancy, excessive blood loss, and insufficient nutritional or digestive uptake results in organic and systemic shortcomings. Established *in situ* techniques, such as EDX-ray spectroscopy, are not sensitive enough to analyze trace metal distribution and the quantification of metal images is difficult.

**Methods:**

In this study, we developed a quantitative biometal imaging technique of human liver tissue by laser ablation inductively coupled plasma mass spectrometry (LA-ICP-MS) in order to compare the distribution of selected metals in cryo-sections of healthy and fibrotic/cirrhotic livers.

**Results:**

Most of the metals are homogeneous distributed within the normal tissue, while they are redirected within fibrotic livers resulting in significant metal deposits. Moreover, total iron and copper concentrations in diseased liver were found about 3-5 times higher than in normal liver samples.

**Conclusions:**

Biometal imaging *via* LA-ICP-MS is a sensitive innovative diagnostic tool that will impact clinical practice in identification and evaluation of hepatic metal disorders and to detect subtle metal variations during ongoing hepatic fibrogenesis.

## Introduction

Hepatic fibrosis, cirrhosis and hepatocellular carcinoma are characterized by a drastic increase of extracellular matrix proteins comprising collagens, glycoproteins, proteoglycans and hyaluronan [1], [2]. The pathogenesis is triggered by various noxious agents (e.g. alcohol, drugs, viruses, and parasites), cholestasis, autoimmune reactions, metabolic diseases, or other exogenous or endogenous stimuli [1], [2]. In addition, the overload with metals or trace elements that originates from hereditary disorders, nutritional oversupply, or occupational exposure is potentially causative for the pathogenesis of liver diseases. Hereditary hemochromatosis for example is the most common inherited disease of iron metabolism that results in toxic accumulation of iron in parenchymal cells of the liver [3]. This disease is most often associated with a polymorphism within the *HFE* gene that is quite common among white people resulting in an amino acid exchange (i.e. C282Y). Homozygous carriers usually have increased serum transferrin saturation levels and increased serum ferritin levels. In addition, non-*HFE* hepatic iron overload diseases originate from mutation in other genes such as transferin receptor 2, hepcidin, hemojuvelin, and ferroportin [4]. Since these genes influence net iron concentration by affecting the synthesis and activity of the peptide hormone hepcidin that represents the master regulator of iron homeostasis, respective mutational alterations most often give rise to elevated concentrations of circulating iron and thus overload occurs. Conversely, iron deficiency resulting from decreased iron uptake, low absorption, rapid growth, pregnancy, and blood loss provoked by frequent blood donation or heavy menstrual periods, leads to anemia impairing proper body function.

Copper is a unique essential trace element playing an important function as a cofactor for a number of cellular processes [5]. Copper homeostasis is regulated by copper uptake in the gastrointestinal tract, distribution through the body, and excretion mainly into the bile. At the cellular level two copper transporters namely CTR1 and CTR2 regulate the intake, while copper efflux occurs *via* the ATP-dependent pumps ATP7B and ATP7A [6]. Hepatic copper accumulation can result from increased copper intake, inherited metabolic defects or from a reduced biliary excretion of copper. Wilson disease (WD) is an autosomal recessive disease caused by mutations of the WD gene *ATP7B* resulting in copper accumulation in different tissues. In liver, ATP7B is mainly expressed in hepatocytes and its loss is the basis for reduced hepatic biliary copper excretion and reduced incorporation of copper into ceruloplasmin [7]. Affected patients display hepatic and neurological disease with yet poorly understood pathomechanisms. In line, *Atp7b* ^-/-^ mutant mice display a gradual accumulation of hepatic copper inducing gross anatomic liver abnormalities in older mice with an irregular liver surface with protruding regenerative nodules of different sizes indicative for cirrhosis [8].

These examples underline that body metal homeostasis is crucial and metal deficiencies or dramatic build-ups should be prevented to avoid hepatic or other organic impairments. Therefore, indicative, non-invasive serum parameters (e.g. serum transferrin concentration and saturation, ferritin, haemoglobin, hematocrit, serum ceruloplasmin) and genetic testing of causative genes is widely propagated in diagnosis to identify metal disorders or predisposition for respective diseases. Moreover, the performance of liver biopsy to measure or stain *in situ* metal content in combination with scanning electron microscopy with energy-dispersive X-ray analysis (SEM-EDX) is traditionally the confirmatory test in cases in which the non-invasive markers suggest elevated metal concentrations. Unfortunately SEM-EDX is often not sensitive enough for trace element imaging. However, liver heterogeneity can result in wrong analytical data). Therefore, novel imaging techniques that provide information about the regional distribution of individual metals or groups of metals combined with a diagnostic score system further providing information about the extent of hepatic insult are urgent required.

Although potentially not causative, the outcome of hepatic fibrosis is also significantly modulated by variations in hepatic metal distribution and concentrations. Hepatic iron overload for example has been described in chronic hepatitis C as a cofactor affecting fibrosis progression and hepatic iron deposits were found in well-compensated chronic hepatitis B infection, especially in patients that were co-infected with hepatitis D virus [9]. Likewise, severe copper deficiency exacerbates liver injury and liver fibrosis in rats that underwent experimental bile duct ligation surgery [10], again demonstrating that variations in individual metal concentrations might not only be the result of ongoing hepatic fibrogenesis but are further important modulators in disease progression. Therefore, there is no doubt that accurate method allowing to measure hepatic metal concentrations or to detect metals deposits are of important diagnostic relevance not only in determination liver insult but further to predict overall disease outcome.

Magnetic resonance imaging (MRI) allows the mapping only of selected single metals (such as iron) in organs [11], but the quantification is often difficult [12]. Novadays, laser ablation inductively coupled plasma mass spectrometry (LA-ICP-MS) techniques with multi-element capability and high spatial resolution are herein discussed has been established to a new generation of sensitive analytical tools for imaging of metals in biological tissue.

Recently, LA-ICP-MS imaging technique has been applied as a reference standard method to validate the results from quantitative gadolinium enhanced MRI technique [12]. Although LA-ICP-MS imaging has been introduced in diagnosis of metal disorders in brain [13]–[16], this innovative technique has not been used in quantitative bioimaging of metals in human liver. A first report from Feldmann’s working group from 2003 demonstrated for copper and zinc in sheep liver sections the capability of LA-ICP-MS for elemental mapping [17]. In another previous study, LA-ICP-MS imaging revealed that *staphylococcal aureus* liver abscesses from normal mice are enriched in Ca^2+^, while these abscesses are devoid of detectable Zn^2+^ and Mn^2+^ [18]. These studies are encouraging and show that LA-ICP-MS is indeed a methodology that might be suitable to detect delicate metal alterations in liver that are the cause or a consequence of organ alteration or dysfunction.

The aim of the present study is to develop a novel quantitative imaging technique for trace metals and to characterize the distribution of various metals in cryo-sections of normal, fibrotic and cirrhotic human liver tissues. We demonstrate that most of the metals analyzed are regularly distributed within normal liver tissue, while they are redirected within fibrotic and cirrhotic livers resulting in defined deposits suggesting that this method is not only valuable in diagnosis of common metal disorders but also adequate to determine subtle changes during ongoing liver fibrogenesis.

## Materials and Methods

### Patients and informed consent

Liver samples were taken from internal tissue banks of the RWTH University Hospital Aachen or the University Hospital Regensburg in which liver tissue was used for experimental purposes that was acquired either from biopsies for routine clinical purposes or explants of cirrhotic livers that were obtained during liver transplantation. Each patient from which material is stored provided written informed consent and the study was approved by the local ethics committee at the RWTH Aachen University and the University of Regensburg. Liver fibrosis/cirrhosis was diagnosed mainly on the grounds of liver routine histology. Fibrotic/cirrhotic tissue samples used in this study were obtained from five independent patients (D1 – D5). Secluded tissue from tumour-free margins of resected hepatic metastasis within normal liver parenchyma (N1 – N5) served as controls.

### Immunohistochemical characterisation of liver specimens

After being fixed in 4% buffered formalin for 24 h, the liver tissue was embedded into paraffin wax. A histological semi-quantitative examination of the liver was performed on sections after standard Sirius Red and H & E stains. Liver sections were stained with an anti-human α-SMA antibody using standard procedures. To assess the degree of fibrosis, a standard Ladewig staining was performed and the severity of liver biopsy lesions and overall immunological activity were graded and staged according to routine scoring guidelines of Desmet and Scheurer [19]. Patients with cirrhosis were stratified according to the Child-Pugh score. The characteristics of the biopsies used in this study are given in Table 1.

**Table 1:** Characteristics of biopsy samples used in this study.

No.	Internal Number / Origin	Remarks / Desmet-Scheuer Score	Etiology of disease
N1	NG237 / Aachen	no fibrosis/ 0	liver metastasis from colon adenocarcinoma
N2	NG238 / Aachen	no fibrosis/ 0	liver metastasis from breast adenocarcinoma
N3	NG265 / Aachen	no fibrosis/ 0	hepatocellular carcinoma
N4	27 (normal) / Regensburg	no fibrosis/ 0	no known disease history
N5	29 (normal) / Regensburg	no fibrosis/ 0	slight signs of steatosis due to alcohol abuse
D1	NG205 / Aachen	mild fibrosis, portal extracellular matrix deposits/ 1	liver metastasis from colon adenocarcinoma; fibrosis of unknown origin
D2	NG241 / Aachen	cirrhosis/ 4	hepatocellular carcinoma; cirrhosis in chronic HCV infection
D3	NG264 / Aachen	cirrhosis/4	hepatocellular carcinoma; cirrhosis of unknown origin
D4	8 (cirrhosis) / Regensburg	cirrhosis/ 4	cryptogenic cirrhosis, burned-out NASH
D5	23 (cirrhosis) / Regenburg	cirrhosis/ 4	cryptogenic cirrhosis, burned-out NASH

### Scanning Electron Microscopy with EDX analysis (SEM- EDX)

Slices of 20 to 50 µm thickness were obtained from paraffin-embedded liver tissue, sputtered with a conductive silver paint to make contact on the surface, carbon-coated in a rotary evaporator, and then maintained in desiccators to prevent air-contact before analysis. Analysis of element content by EDX was rapidly performed within two days of preparation using an environmental scanning electron microscope (ESEM XL 30 FEG, FEI, PHILIPS, Eindhoven, The Netherlands) in a high vacuum environment operating at 15 to 20 kV. The slices were imaged by back-scattered electrons and analysed for the elemental composition of the element present. For quantitative analysis of the element concretions, the contributions of the C-coating and the embedding resin containing C, O and trace of Cl were computer-based subtracted from the quantitative data of each spectrum.

### Metal imaging of liver tissue section by laser ablation inductively coupled plasma mass spectrometry (LA-ICP-MS)

A quadrupole-based inductively coupled plasma mass spectrometer (ICP-MS, XSeries 2, Thermo Scientific, Bremen, Germany) coupled to a laser ablation system (NWR 213, New Wave Research, Fremont, CA, USA) was used to study elemental distributions in tissue sections of human livers (30 µm thickness). Laser ablation of biological tissue was performed using a focused Nd:YAG laser beam in the scanning mode (wavelength 213 nm, repetition frequency 20 Hz, laser spot diameter 60 µm, scanning speed 60 µm s^-1^, laser fluency 0.24 J cm^−2^). The ablated material was transported by argon gas (as carrier gas) into the inductively coupled plasma (ICP). The ions formed in the atmospheric pressure ICP were extracted in the ultrahigh vacuum mass spectrometer via a differential pumped interface, separated in the quadrupole mass analyzer according to their mass-to-charge ratios and detected by an ion detector. No reference standard materials for quantification of metals in human liver were available. Therefore, SRM NIST 1577b (bovine liver) was used as standard reference material. The trace metal concentrations in the samples were determined by single point calibration using this SRM. Moreover, the selection of internal standard element is the important part for LA-ICP-MS analyzing. The appropriate internal standard element was chosen to correct for plasma instabilities and sample-to-sample variations in the ion signal intensity. In this work, sulphur was used as an internal standard element for all the analyses because sulphur in human livers present rather a homogeneous distribution and provide constant concentration (see below). The human liver tissue and the NIST standard reference material deposited on glass slide were mounted in the laser ablation chamber to perform LA-ICP-MS imaging of sample and standard reference material under identical experimental conditions. Mass spectrometric measurements by LA-ICP-MS for imaging of liver tissue were performed by line scanning ablation (line by line) with a focused laser beam under the optimized experimental parameters given in Table 2. The experimental parameters of LA-ICP-MS were optimized with respect to the maximum ion intensity of ^63^Cu^+^ using a SRM 1577b bovine liver standard. To validate the metal ion images, two isotopes of the same element were analyzed, whenever possible. From the continuous list of raw pixel values elemental images were reconstructed using the IMAGENA LA-ICP-MS Image Generation software created at Forschungszentrum Juelich [20]. Trace metal concentrations were calculated from ion intensities averaged throughout freely drawn regions of interest (ROIs) within ion intensity images using PMOD version 3.0 (details see www.pmod.com).

**Table 2:** Optimized experimental parameters used for LA-ICP-MS imaging of human liver samples.

ICP mass spectrometer	ICP-QMS, Thermo XSeries II
Rf power	1450 W
Cooling gas flow rate	16.0 L min^-1^
Auxiliary gas flow rate	0.7 L min^-1^
Carrier gas flow rate	1.0 L min^-1^
Dwell time	20 ms
Extraction lens potential	3400 V
Mass resolution (m/Δm)	300
Scanning mode	peak hopping
Analysis time per liver sample(10 mm×10 mm)	4 hours
Laser ablation system	New Wave (NRW213)
Wavelength of Nd:YAG laser	213 nm
Laser fluence	0.24 J cm^-2^
Repetition frequency	20 Hz
Laser spot size	60 µm
Scan speed	60 µm s^-1^
Ablation mode	line scan

## Results

### Analysis of liver specimen by SEM-EDX

SEM-EDX is used for chemical analysis and determination of the elemental composition of microscopic particles or regions within a sample by *in situ* measuring the energy and intensity distribution of X-ray signals generated by a focused electron beam on the specimen (i.e. biopsy). It relies on the investigation of an interaction of some source of X-ray excitation and the sample resulting in a unique set of peaks on the X-ray spectrum that is characteristic for each element. In brief, the necessary equipment for this technique is based on an excitation source that transmits an electron beam, an X-ray detector that converts X-ray energy into voltage signals, a pulse processor and an analyzer that processed the signals and matches them to individual atoms. Based on the fact that this technique allows discriminating of metals, this technique is used to corroborate or even score metal disorder in organ tissue.

**Figure 1:**
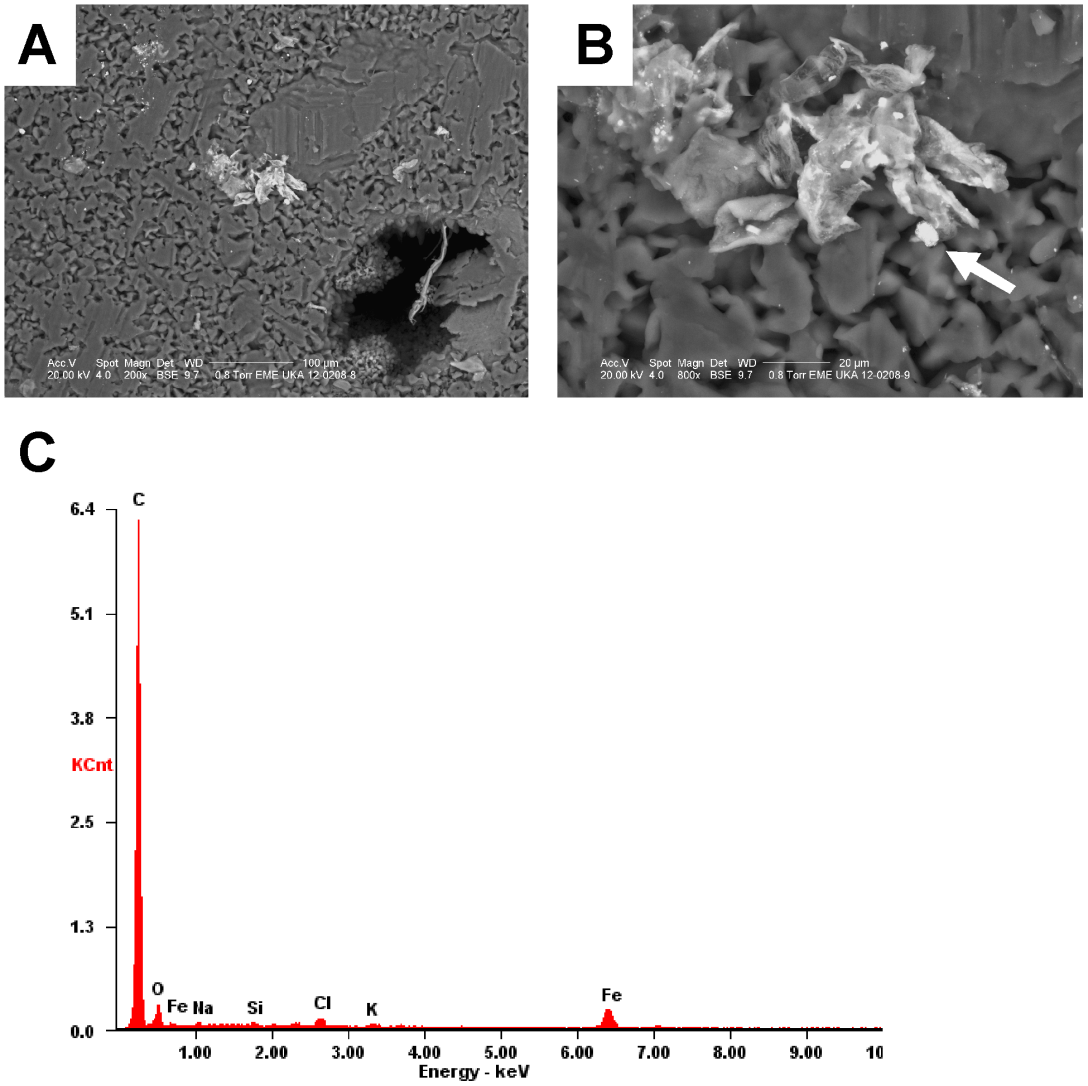
Representative SEM/EDX microanalysis in liver. (A) SEM overview of a paraffin embedded liver specimen from a patient (N1) suffering from fibrosis. **(B)** The metal deposit that was subsequently analysed by EDX is marked by a white arrow. **(C)** Resulting elemental EDX microanalyses of the marked mineral crust in **(B)**. The peaks in the spectrum are labelled with the EDX line of the corresponding element.

When we applied this method to non-WD patients suffering from liver fibrosis or cirrhosis, we noticed that although it allows the identification of individual metal deposits (Figure 1), it was unsuitable to discriminate between normal, fibrotic or cirrhotic liver tissue. Moreover, the identification of metals by SEM-EDX is somewhat critical because several elements have overlapping peaks and data interpretation is somewhat enigmatic and only possibly by highly experienced investigators.

### LA-ICP-MS analysis of liver tissues

The introduction of LA-ICP-MS technology has recently been successfully applied as a powerful imaging (mapping) technique to produce quantitative images of detailed regionally specific element distributions in thin tissue sections of human or rodent brain [21].

**Figure 2:**
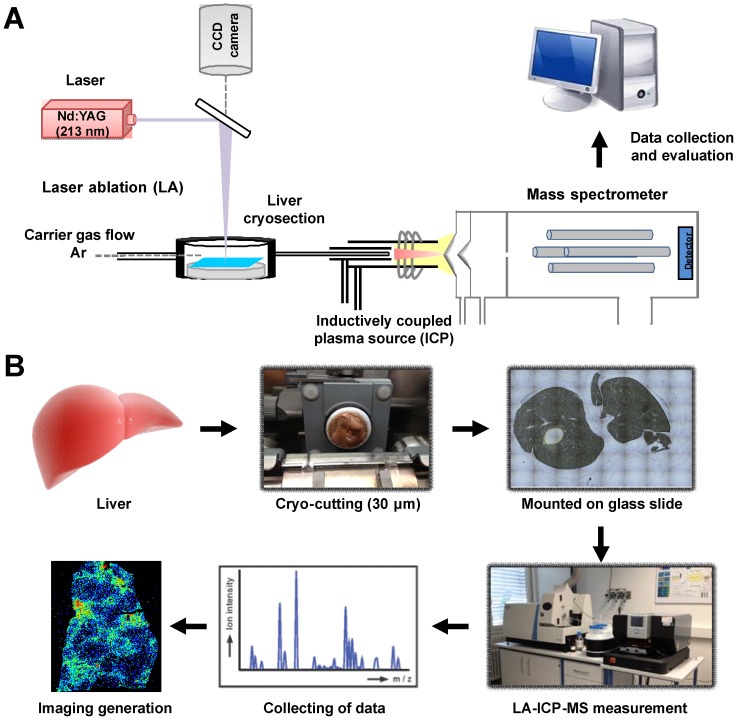
Principle and workflow of LA-ICP-MS for hepatic metal imaging. **(A)** Principle and **(B)** Workflow of imaging mass spectrometry from sample preparation of thin section by cryo-cutting, *via* the LA-ICP-MS measurement procedure by scanning of thin tissue section (line by line), acquisition and evaluation of analytical data including quantification using single point calibration (NIST SRM 1577b bovine liver).

In an attempt to use this methodology for analysis of liver tissue, we established a protocol that allows quantitative metal imaging of liver tissue. In the final workflow, liver tissue is cryo-sected into specimen of 30 µM thickness and mounted on glass slides (Figure 2B). Mass spectrometric measurements is then performed in the LA-ICP-MS by line scanning ablation (line by line) with a focused laser beam under the optimized experimental parameters given in Table 1.

**Figure 3:**
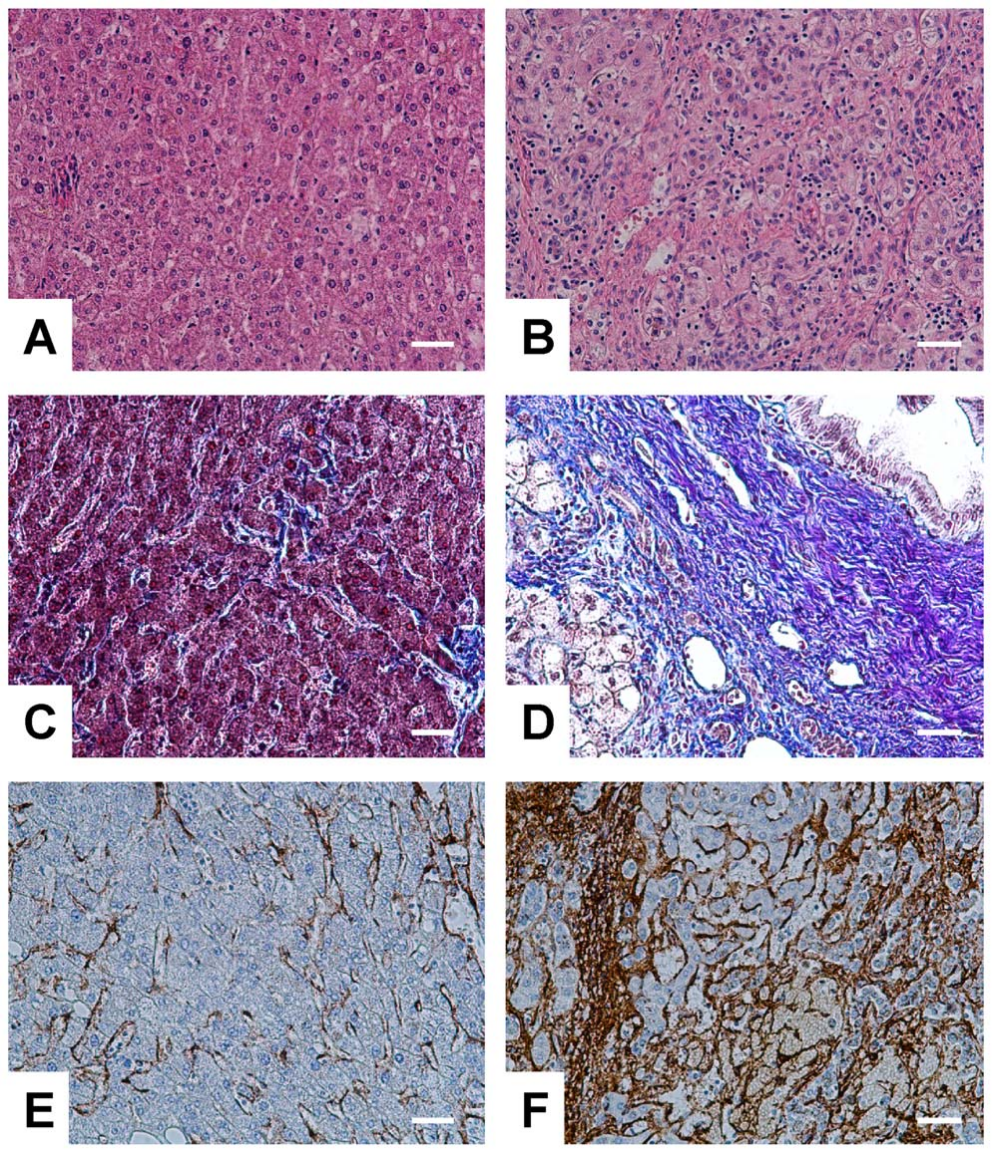
Representative immunohistochemical analysis of liver sections of patients enrolled in this study. (A, C, E) Normal (N1) and (B, D, F) cirrhotic liver tissues (D2) were stained with (A, B) hematoxylin and eosin, (C, D) Ladewig, or (E, F) probed with an antibody specific for α-SMA. The space bar in each figure represents 100 µM each.

To evaluate the LA-ICP-MS imaging technique in liver specimen and to further analyse if the concentration or distribution of individual metals is altered in fibrotic or cirrhotic liver tissue, we thought to apply this imaging technique in a small set of histological well-characterized samples from normal, fibrotic and cirrhotic liver tissue (Figure 3).

### Trace metal imaging and quantification in selected liver sections

**Figure 4:**
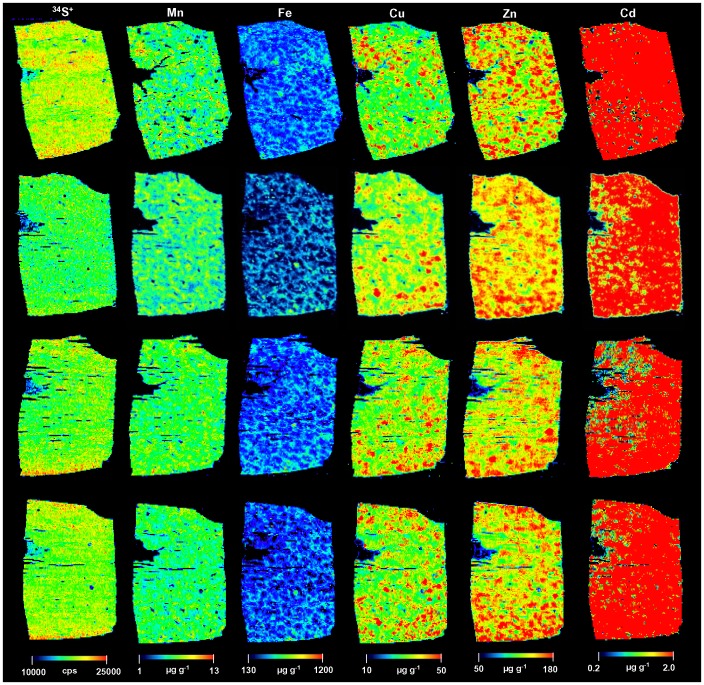
Reproducibility of imaging of elements in liver tissue sample. Representative spatial distribution of elements of interest (S, Mn, Fe, Cu, Zn, and Cd) in four adjacent sections from the same human liver tissue is depicted.

The reproducibility of the LA-ICP-MS imaging technique for the analysis of 20 µm thin cross section of human brain tissues is already well studied [22]. 3% reproducibility had been observed for homogeneous tissues (thin cross sections of matrix-matched laboratory standards) and 5%–7% reproducibility for inhomogeneous tissue. Moreover, the applicability of LA-ICP-MS technique was demonstrated in Parkinson mouse brain 14 µm sections [23], [24]. The reproducibility of 5% for Fe and Cu, 12% for Zn, and 11% for Mn was presented. In our study, the replicate measurement of 4 adjacent sections of the same human liver tissue (N3) were scanned using optimum condition of LA-ICP-MS in order to determine the reproducibility (see Figure 4). The reproducibility values calculated were 9% for S, 10% for Mn, 14% for Fe, 6% for Cu, 4% for Zn and Cd. Since sulphur is an abundant element showing an overall homogenous distribution in liver (see Figure 4, left panel), we used this non-metal as an internal standard.

**Figure 5:**
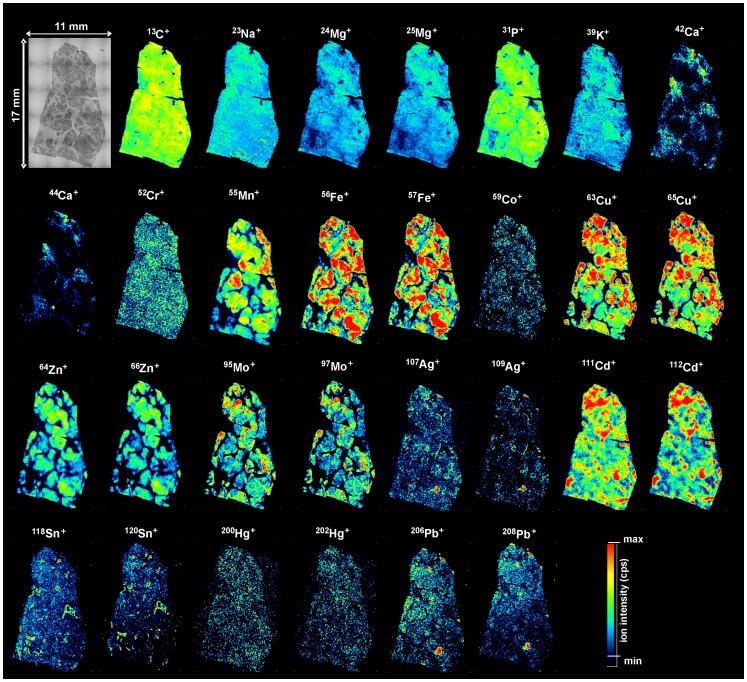
LA-ICP-MS imaging of essential and toxic metals and carbon in hepatic cirrhosis. Representative LA-ICP-MS maps of C, Na, Mg, P, K, Ca, Cr, Mn, Fe, Co, Cu, Zn, Mo, Ag, Cd, Sn, Hg, and Pb isotope as detected in cirrhotic human liver sample (D3).

Figure 5 shows LA-ICP-MS images of the detected elements (C, Na, Mg, P, K, Ca, Cr, Mn, Fe, Co, Cu, Zn, Mo, Ag, Cd, Sn, Hg, and Pb) in a fibrotic liver sample (N3). Ca, Mn, Fe, Cu, Zn, Mo, Cd, were detected with a higher abundance in fibrotic zones compared to normal zones, whereas the ion intensity measured of C, Na, Mg, P, K, Cr, Co, Ag, Sn, Hg, and Pb were not different between fibrotic and normal zones. This picture presents the potential of LA-ICP-MS to investigate the distribution of various essential and toxic elements. However, no quantification of some elements (C, P, Mo, Sn, and Hg) was possible due to the lack of certified concentrations in the bovine liver standard reference material used in this study.

**Figure 6:**
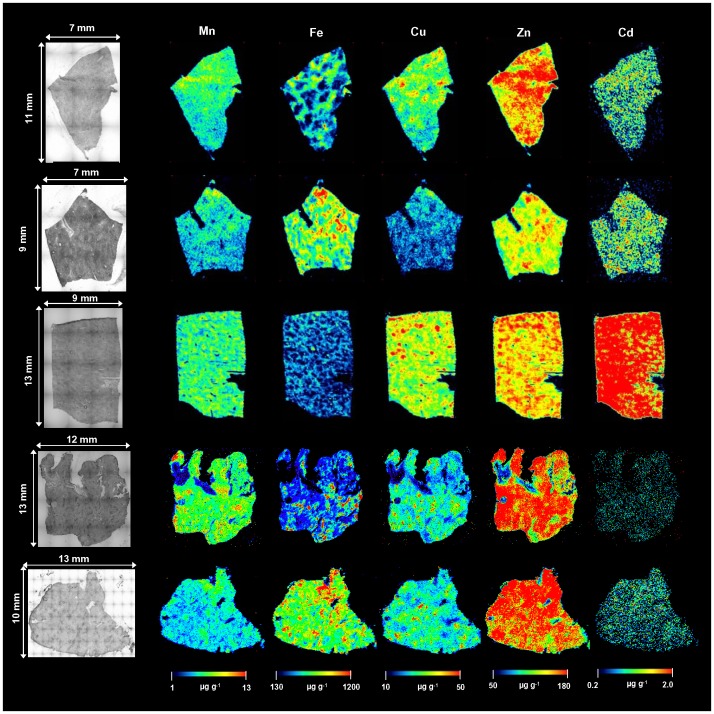
Selected LA-ICP-MS images in control liver samples. Images of Mn, Fe, Cu, Zn and Cd of control human liver samples (N1-N5) measured by LA-ICP-MS.

**Figure 7:**
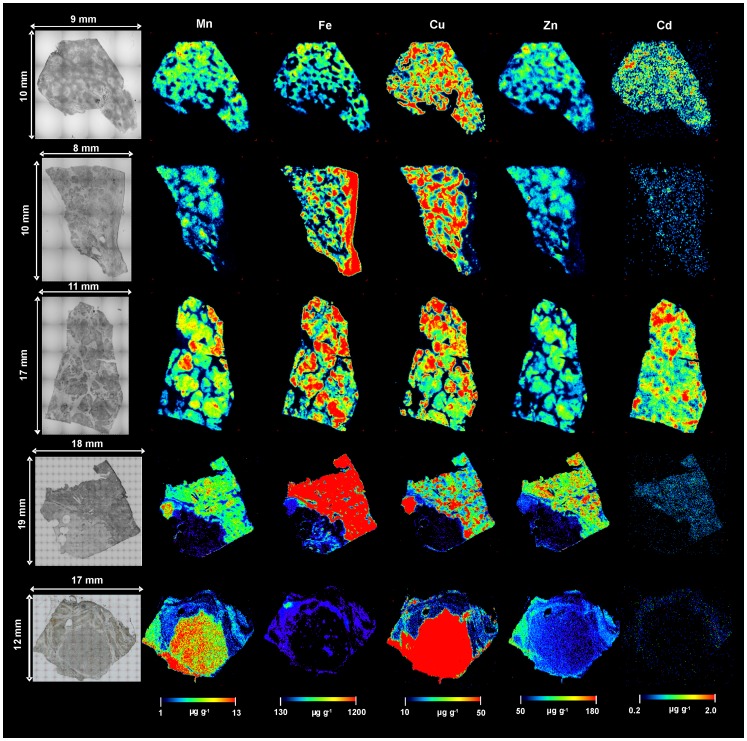
Selected LA-ICP-MS images in diseased liver. Images of Mn, Fe, Cu, Zn and Cd of fibrotic (D1) and cirrhotic human liver samples (D2-D5) measured by LA-ICP-MS.

Quantitative bioimages of Mn, Fe, Cu, Zn, and Cd of control and diseased (fibrotic and cirrhotic) human liver samples are illustrated in Figure 6 and Figure 7, respectively. In the case of control liver samples, Mn, Cu, Zn, and Cd images are homogeneous distributed. For Fe images, the hexagonal structure was found clearly in N1 and N3 control liver sections. These hexagonal shapes are corresponding to the structure of human liver lobule and reflect the typical histological pattern of iron deposition in the normal liver. As shown in the Fe image, Fe was distributed predominantly around the rim of lobule structure. Fe distribution presented a decreasing gradient from the rim area to the central area. However, these hexagonal structures cannot be investigated in disease liver tissue section (Figure 7).

The bioimaging approach of metals in fibrotic/cirrhotic liver tissue sections by LA-ICP-MS revealed inhomogeneous distribution of Mn, Fe, Cu, Zn, and Cd. Very low concentrations of metals were observed in fibrous septa areas when compared to nodules areas. Moreover, the concentrations of Fe and Cu in all disease liver (Figure 7) were significant higher than all control liver section (Figure 6). In contrast, Zn concentrations in control liver samples were higher than in disease liver. In comparison with the high Fe content region (mark within circle area), it’s was found low concentration of Cu (Figure 7).

**Figure 8:**
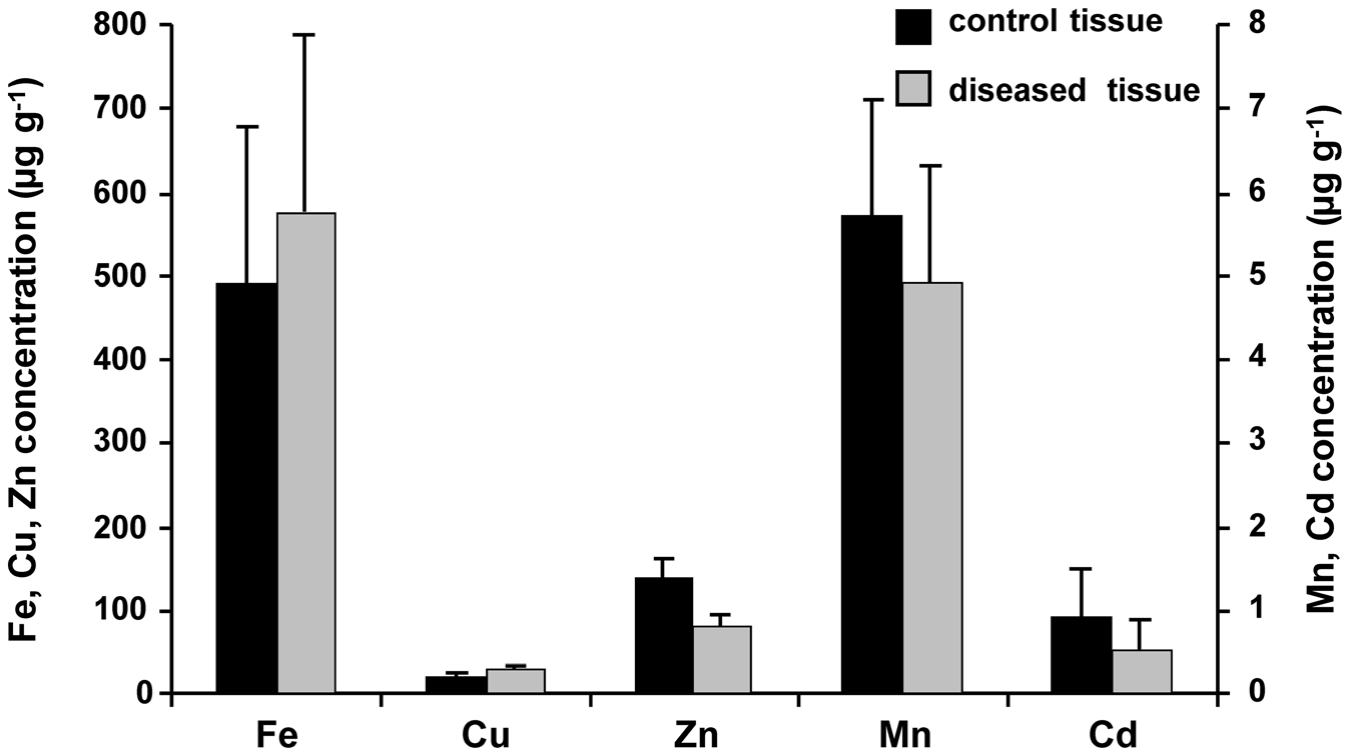
Quantitative metal distribution in liver samples. Comparative measurement of Mn, Fe, Cu, Zn, and Cd concentration in sections through the control (n  =  5) and diseased (fibrotic/cirrhotic) (n  =  5) liver tissue.

Figure 8 compares the average of trace metal concentrations measured in the control liver tissue (N1–N5) and disease liver tissue (D1–D5). The average concentrations of Mn, Fe, Cu, Zn, and Cd were 5.8, 493, 23, 140, and 1 µg g^-1^ in control; were 4.9, 576, 35, 80, and 0.5 in disease (fibrotic/cirrhotic) liver samples. In the case of Mn and Cd, the different concentrations between control and disease are not significant. On the other hand, the concentration of Fe, Cu, and Zn are quite different between the control and disease liver. Fe and Cu concentrations in diseased liver are higher than in normal liver samples, while Zn concentrations are decreased in diseased samples suggesting that these changes can be used to predict the changing of liver state from healthy to diseased liver.

## Discussion

Hereditary liver diseases resulting in metal intoxication are reasons for morbidity and mortality worldwide [25], [26]. In addition, various liver diseases including both alcoholic and non-alcoholic steatohepatitis are associated with increased iron deposits [27], [28]. Furthermore several trace elements are known to influence the outcome of hepatitis virus infections [29]. Although there have been great advancements in the establishment of novel genetic test systems and measurements of serum parameters that are indicative and suitable for identification of respective diseases or intoxications, metal stains in liver sections and other techniques like SEM-EDX measurements are traditionally the confirmatory diagnostic tests for hepatic metal poisoning and is one the first sight attractive because (i) the detectors have high sensitivity due to a wide solid angle collecting the X-rays emitted from the sample, (ii) the entire chemical composition of the analyzed specimen can be observed without wavelength scans, and (iii) analysis time are rather small [30]. However, the accuracy of techniques that are based on electron-microscopic analysis of liver sections such as SEM-EDX is limited by several factors. First the analysts performing the SEM have a bias and restrict their analysis to material deposits that are visible. Second several elements in EDX have overlapping peaks that under certain constellations does prevent precise assignment of signals to element. Third quantification of individual element concentration is impossible or only hardly to achieve. Finally, the accuracy of the spectrum can also be affected by the nature of the sample *per se* in that way that inhomogeneous samples result in inadequate or dissimilar excitations.

X-rays can be generated by any atom in the sample that is sufficiently excited by the incoming beam. These X-rays are emitted in any direction, and so they may not all escape the sample. The likelihood of an X-ray escaping the specimen, and thus being available to detect and measure depends on the energy of the X-ray and the amount and density of material it has to pass through. This can result in reduced accuracy in inhomogeneous and rough samples.

All these limitations of SEM-EDX measurements become evident in our experiment that is depicted in Figure 1 demonstrating that this method is not sensitive enough to illustrate trace metal distribution or even quantify individual metals in the analyzed liver specimens.

LA-ICP-MS on the other side is one of the most powerful and sensitive techniques for the analysis of a variety of solid samples because it provides good sensitivity for major, minor, trace, and ultra-trace elements with high sample throughput and without sample preparation which generates a large amount of waste as occurring in solution-based techniques [14]. Several years ago, the imaging analysis for biometal concentrations in human tissues obtained either by biopsy or postmortem was becoming an increasingly important function of the clinical laboratory.

The use of matrix matched reference material for quantification of analytical data is important because the ablation yield varies with the sample matrix. However, an appropriate matrix-matched standard reference material that for metal in human liver is not available. The most common quantification method for tissue in LA-ICP-MS is external calibration utilizing matrix-matched standards [24]. In the ongoing project, suitable matrix-matched tissue standards with well-defined concentrations of the biometals of interest were created and used for the calibration of the LA-ICP-MS technique. Several independent other studies have shown that this one point calibration method also known as offset-only calibration gives a reasonable accuracy in quantification of various metals in LA-ICP-MS [12], [17], [31], [32].

Human liver consists of two main lobes, left and right, which overlap slightly. Each lobe contains lobules that are the building blocks of the liver that are six-sided structures or hexagonal shapes. As shown in Figure 6, Fe-rich regions are found around the rim of hexagonal shape. In contrast, the concentrations of Fe become lower at the central of structure. Our results also support the previous finding, which investigated the distribution of Fe in liver tissue. Kinoshita and coworkers have been reported distribution in the lobule of human liver by synchrotron radiation X-ray fluorescence (SRXRF) microscopy [33]. In their report, Fe was distributed predominantly in periportal hepatocytes in the normal liver in a decreasing gradient from the periportal area (rim of hepatocyte) to the perivenous area (central of hepatocyte).

Mn is one of the important trace biometal, which can be detected in human liver. Mn is secreted in bile and concentration increase in cholestatic liver disease. The manganese content of the liver is very high as compared to its concentration in serum [34]. It has also been demonstrated that the liver has an important role in the excretion of manganese. Previous findings reported the increasing of whole blood Mn concentrations in patients with liver cirrhosis [35], [36]. Moreover, they suggested that in cirrhosis of the liver failure of biliary excretion of Mn leads to overload and subsequent cerebral accumulation of this metal. Mn accumulates in mitochondria, a major source of superoxide, which can oxidize Mn^2+^ to the powerful oxidizing agent Mn^3+^ [37]. Oxidation of important cell components by Mn^3+^ has been suggested as a cause of the toxic effects of it. For our results, in fibrotic/cirrhotic tissue samples (Figure 7), Mn was increased to higher concentration in fibrotic and cirrhotic zones compared to non-tumor zones. However, the average concentrations of Mn in control liver and disease samples (Figure 8) are not significant different.

In the animal model of liver fibrogenesis, iron acts as a “co-factor” of fibrogenesis [38]. Within the liver, iron related oxidative stress can lead to fibrosis and ultimately to cirrhosis [39]. When present in excess within the cell (hemochromatosis), iron can be toxic due to its ability to catalyze the formation of damaging radicals *via* a chemical reaction known as the Fenton reaction, which promote cellular injury and cell death. The results shown in this study could be in agreement with results from previous studies. In the control livers, the distributions of Fe are quite homogeneous. In contrast to disease livers, higher concentration of Fe was found in fibrotic or cirrhotic area when compared to normal area. For fibrotic liver, Fe concentration is higher than control tissue (Figure 8). However, the different between these values are not significant. In contrast to fibrotic tissue, the average concentrations of Fe in cirrhotic liver samples are higher than control liver about three times.

Not only Fe overload, the accumulation of too much Cu in WD is able to catalyze the development of liver disease. The major physiologic aberration is excessive absorption of copper from the small intestine and decreased excretion of copper by the liver. WD is an inherited metabolic disorder characterized by accumulation of copper in the tissue leading to progressive hepatic damage in the liver and the other organs [40], [41]. With progressive parenchymal damage, fibrosis and subsequently cirrhosis can be developed. In early stages of the disease, copper is mainly in the cytoplasm bound to metallothionein and is not histochemically detectable; in later stages, copper is found predominantly in lysosomes [41]. The amount of copper varies from nodule to nodule in cirrhotic liver and may vary from cell to cell in pre-cirrhotic stages. The absence of histochemically identifiable copper does not exclude WD, and this test has a poor predictive value for screening for WD. By use of LA-ICP-MS, we found inhomogeneous distributions in all fibrotic/cirrhotic liver sections analyzed. The average concentrations of fibrotic and cirrhotic are 204, and 210 µg g^-1^, and 39.4 µg g^-1^ for control tissue samples. With these data, Cu is the important element that relates to the stage of liver failure and therefore used as an indicator for liver damage.

Zn is necessary for normal liver function and, the liver plays a central role in Zn homeostasis [42], [43]. The liver is important for the regulation of zinc homeostasis, while zinc is necessary for proper liver function. It has long been speculated that Zn has a protective effect against liver fibrosis [42], [44]. In our study, Zn concentrations in liver tissue were decreased from control (319 µg g^-1^), fibrosis (223 µg g^-1^), and cirrhosis (192 µg g^-1^), respectively. Data obtained from our and previous studies suggest that ongoing liver disease correlates with reduced Zn levels. These findings fit quite well with recent experimental observations showing that Fe and Cu act in an antagonistic fashion and iron is an important factor triggering copper deficiency in liver [45].

Cadmium is a highly toxic element present in food and water. It can deposit in the liver and kidney and strongly binds to macromolecules in the intracellular compartment and influences the metabolism of Fe, Ca, Zn, Mn and Cu [46], [47]. Therefore, the overloading of Cd in liver may be disturbed the protective function of Zn to liver disease. However, Cd concentrations of control samples in our study are slightly higher than in fibrotic and cirrhotic samples. This result may be due to the fact that Cd concentration in liver tissue varies depending on many factors. The major route for cadmium intake for non-smoker is ingestion of cadmium in foodstuffs of natural origin or from the use of phosphate fertilizers and sludge on agricultural soils. Smokers have elevated blood and tissue concentrations of cadmium from cigarette smoke. As shown in Figure 8, the average concentration of the toxic trace metal Cd is not higher than 1 µg g^-1^ in all tissue samples.

Considering all known hepatic diseases that are caused, promoted or modulated by accumulation of metals, there is a mandatory need for the establishment and evaluation of precise non-invasive metal imaging methods for diagnostics. In recent years, many studies have demonstrated the possibility of performing hepatic iron quantification with magnetic resonance imaging technology. However, a consensus has not been reached yet regarding the technique or the possibility to reproduce the same method of calculus in different equipments and intensive discussion for the establishment of consensus guidelines for clinical practice of hepatic iron quantification by magnetic resonance imaging techniques are conducted [48]. Since recent experimental and clinical studies have further demonstrated that different metals act as strong modifiers that influence the outcome of hepatic fibrogenesis [10], [49], it is obvious that the quantitative trace metal determination in the diagnosis of disease progression will potentially become more important in future. The demonstration that LA-ICP-MS imaging representing one direct solid sampling technique for major, minor and trace element analysis is suitable for biometal analysis in normal and disease liver will therefore enrich the spectrum of diagnostic options. It allows to parallel measure the metal content in cryo-sections that become available in routine biopsy that still represent the gold standard for accurate assessment of the degree of fibrosis or presence of cirrhosis.

It would be an absolute goal to further analyze the sub-lobular and sub-cellular distribution of signals regarding different metals in both normal and disease conditions. In our study we noticed that the signals of several metals are more punctuated than others. However, the precise mapping of individual metals to cellular subpopulation of the liver is a complex venture and must be addressed in future studies. There are however already pioneering studies available that have already precisely co-localized aggregates and mercury deposits within cellular subpopulations of the liver [50]. Such studies would be fundamental for understanding the pathophysiological relevance of individual hepatic cells including Kupffer cells, hepatocytes, sinusoidal endothelial cells, hepatic stellate cells, and Pit cells (or even hepatic stem cells) in any kind of metal-induced liver disorders.

In summary, all these findings indicate that LA-ICP-MS is a novel powerful and innovative analytical technique that will have tremendous impact on diagnostics of metal disorders in liver. The fact that the obtained metal concentrations in liver tissue were the same in specimen that were obtained from two different hospitals (Aachen and Regensburg) further demonstrates that the results obtained in LA-ICP-MS are mostly independent from potential pre-analytical differences that may arise from sample collection. Although we presently do not know how individual hepatic disorders impact the distribution and location of individual metals or groups, it is known that metal overload or lack of individual metals may induce or contribute to the pathogenesis of liver disease. Therefore, LA-ICP-MS allowing quasi-simultaneous measurement of all metals and selected non-metals will provide an important add-on to routine diagnosis of liver sections or biopsies.
